# Adsorption of an Ideal Gas on a Small Spherical Adsorbent

**DOI:** 10.3390/nano11020431

**Published:** 2021-02-09

**Authors:** Bjørn A. Strøm, Dick Bedeaux, Sondre K. Schnell

**Affiliations:** 1 Department of Materials Science and Engineering, Faculty of Natural Sciences, Norwegian University of Science and Technology, NTNU, NO-7491 Trondheim, Norway; sondre.k.schnell@ntnu.no; 2 Porelab, Department of Chemistry, Norwegian University of Science and Technology, NTNU, NO-7491 Trondheim, Norway; dick.bedeaux@ntnu.no

**Keywords:** adsorption, nanothermodynamics, small-system, size-dependent, thermodynamics, statistical mechanics, ideal gas, nanoparticles

## Abstract

The ideal gas model is an important and useful model in classical thermodynamics. This remains so for small systems. Molecules in a gas can be adsorbed on the surface of a sphere. Both the free gas molecules and the adsorbed molecules may be modeled as ideal for low densities. The adsorption energy, $U^{s}$, plays an important role in the analysis. For small adsorbents this energy depends on the curvature of the adsorbent. We model the adsorbent as a sphere with surface area $\Omega =4\pi R^{2}$, where *R* is the radius of the sphere. We calculate the partition function for a grand canonical ensemble of two-dimensional adsorbed phases. When connected with the nanothermodynamic framework this gives us the relevant thermodynamic variables for the adsorbed phase controlled by the temperature *T*, surface area Ω, and chemical potential μ. The dependence of intensive variables on size may then be systematically investigated starting from the simplest model, namely the ideal adsorbed phase. This dependence is a characteristic feature of small systems which is naturally expressed by the subdivision potential of nanothermodynamics. For surface problems, the nanothermodynamic approach is different, but equivalent to Gibbs’ surface thermodynamics. It is however a general approach to the thermodynamics of small systems, and may therefore be applied to systems that do not have well defined surfaces. It is therefore desirable and useful to improve our basic understanding of nanothermodynamics.

## 1. Introduction

The objective of the paper is to demonstrate an organized and transparent thermodynamic framework for statistical model development for small systems. The main focus from the thermodynamic side is on the characteristic feature of small systems, namely the effect of size on intensive variables.

We do this by first obtaining the characteristic thermodynamic function for the adsorbed phase from nanothermodynamics as introduced by Hill [1,2,3]. This function is the one that provides us with the fundamental link to statistical mechanics, as it is equal to −kT times the logarithm of the grand canonical partition function. The characteristic function for the adsorbed phase depends on the size Ω of the system. In the macroscopic limit the dependence becomes linear, however when the system is small the subdivision potential measures the deviation from macroscopic behavior. We therefore derive an expression for the subdivision potential in terms of the environment variables, and observe that the differential coefficients of this expression give the dependence of particular intensive properties on size. The size dependence of other intensive thermodynamic properties may then be expressed through thermodynamic relations in terms of the subdivision potential and its derivatives. The close relationship between the subdivision potential and the characteristic feature of small systems is a consequence of the generalization of thermodynamics to small systems, and the framework’s internal structure that follows. This is what we wish to emphasize in this work. For surface problems, the nanothermodynamic approach is different, but equivalent to Gibbs’ surface thermodynamics [1,4,5].

Since the quantities usually referred to as intensive now depend on size, the classical meaning of the term intensive is not appropriate for small systems. It is still a useful term to distinguish thermodynamic quantities, especially since nanothermodynamics is a generalization of classical thermodynamics, and therefore goes over into classical thermodynamics in the macroscopic limit, where this term is ingrained. For a small system quantity, the term intensive is used to describe a quantity that becomes intensive in the classical sense in the macroscopic limit.

We give the thermodynamic framework substance by calculating the subdivision potential of the adsorbed phase. Taking advantage of the simplicity of the ideal gas model, the thermodynamic quantities become more tangible, and it is possible later to gradually increase the complexity from this model to include effects like crowding and cooperativity.

The model consists of an adsorbed phase that is ideal and has an adsorption energy $U^{s}$. The adsorption energy $U^{s}$ will in general depend on the curvature of the sphere, the temperature *T* and the chemical potential μ. However, we want the simplest model possible, in order to make the connection between the nanothermodynamic framework and statistical mechanics as clear and minimal as possible. We therefore consider $U^{s}$ to only depend on Ω, which is consistent with an inert and incompressible adsorbent. The control variables are therefore *T*, Ω, and μ. The dependence on the curvature is characteristic for small spheres.

If the structure of the adsorbent is taken into account, and different crystal structures are considered, this will result in different size dependence for the intensive variables of the adsorbed phase. This is because the surface to volume ratio of the adsorbent becomes a different function of size, and also because edges and corners will have to be considered. This is interesting, but comes at the expense of increased complexity, and is beyond the scope of the article.

## 2. Nanothermodynamics

The thermodynamic system considered here is the adsorbed phase in the context of adsorption of a single component gas on an inert adsorbent. The adsorbent is assumed to be unaffected by the temperature, chemical potential and the adsorbed layer. It functions only as an external field, and is therefore not included in the description of the system. The adsorbed phase is in equilibrium with the gas. The temperature *T*, chemical potential μ, and surface area of the sphere $\Omega =4\pi R^{2}$ form a complete set of independent variables for the adsorbed phase. The surface area Ω determines the radius $R=\sqrt{\Omega /4\pi }$ and the curvature C=2/R. The curvature dependence of the surface tension can therefore be written as a dependence on the surface area Ω. All thermodynamic quantities of the adsorbed phase are functions of *T*, μ, and Ω.

Following Hill [2,3] we consider an ensemble of N independent small systems at temperature *T*, and component chemical potential μ. A complete set of independent variables for the ensemble, with total properties denoted by subscript *t*, may then be taken as the entropy $S_{t}$, area NΩ, the amount of adsorbed component $N_{t}$, and the number or replicas N. We note here that we allow for an independent variation in the size of the small systems, as given by Ω, in addition to the variation in the number of small systems N. This is an essential new feature that allows us to investigate the size of the small system the ensemble represents, and which makes the approach distinct from simply describing a large sample of small systems by conventional thermodynamics.

The characteristic function for the ensemble in terms of the set of independent variables $S_{t}$, Ω, $N_{t}$, and N is the internal energy $U_{t}$ given by

(1) $$dU_{t}=TdS_{t}+\gamma Nd\Omega +\mu dN_{t}+XdN$$

where γ is the ensemble mean surface tension. We will refer to this equation as the Hill-Gibbs equation. The intensive quantities are given by

(2) $$T={\left(\frac{\partial U_{t}}{\partial S_{t}}\right)}_{\Omega ,N_{t},N},\;\;\gamma N={\left(\frac{\partial U_{t}}{\partial \Omega }\right)}_{S_{t},N_{t},N},\;\;\;\mu ={\left(\frac{\partial U_{t}}{\partial N_{t}}\right)}_{S_{t},\Omega ,N}$$

And finally we have the so-called replica energy

(3) $$X={\left(\frac{\partial U_{t}}{\partial N}\right)}_{S_{t},\Omega ,N_{t}}$$

This energy is needed when one ads a replica with a surface area Ω while redistributing the total adsorbed entropy and number of particles over one more replica. Using that $U_{t}$, $S_{t}$, $N_{t}$ are Euler homogeneous in the number of replica N ([2]), i.e., proportional to N, it follows that

(4) $$U_{t}=TS_{t}+\mu N_{t}+XN$$

The internal energy, entropy and number of particles of the adsorbed phase per replica are defined by

(5) $$U\equiv U_{t}/N,\;\;\;S\equiv S_{t}/N,\;\;\;N\equiv N_{t}/N$$

Apart from the entropy *S* the quantities defined in Equation (5) are ensemble mean values of fluctuating extensive quantities ([2], p. 9), ([6], p. 98). For small systems, if a quantity does not fluctuate, but has the same value in every system of the ensemble, it is an environment variable. Here these variables are *T*, Ω and μ. The exception to this rule is the entropy, which is a property of the complete distribution in internal energy and particle number for a single system ([2], p. 9), and is therefore the same for each system. Together with Equation (4) it follows that

(6) $$\hat{\gamma }\Omega \equiv X=U-TS-\mu N$$

This equation also defines $\hat{\gamma }$, the characteristic energy per unit area. Substitution of Equation (5) into Equation (1) and using Equation (6) gives the Gibbs equation for the replicas

(7) dU=TdS+γdΩ+μdN

The important difference of this equation with the usual Gibbs equation is that *U*, *S*, *N* are, for a small sphere, not Euler homogeneous in the surface area Ω, i.e., not proportional to Ω. Differentiating Equation (6) and using Equation (7) we obtain what we call the Hill-Gibbs-Duhem equation

(8) $$d\left(\hat{\gamma }\Omega \right)=-SdT+\gamma d\Omega -Nd\mu$$

It follows from this equation that

(9) $$\begin{array}{ccc} {\left(\frac{\partial \hat{\gamma }}{\partial T}\right)}_{\Omega ,\mu } & = & -\frac{S}{\Omega }\equiv -s,\;\;\;{\left(\frac{\partial \hat{\gamma }}{\partial \mu }\right)}_{T,\Omega }=-\frac{N}{\Omega }\equiv -n\\ {\left(\frac{\partial \left(\hat{\gamma }\Omega \right)}{\partial \Omega }\right)}_{T,\mu } & = & \hat{\gamma }+\Omega {\left(\frac{\partial \hat{\gamma }}{\partial \Omega }\right)}_{T,\mu }=\gamma \end{array}$$

We define the subdivision potential E by

(10) $$E\equiv \left(\hat{\gamma }-\gamma \right)\Omega$$

While the form and physical significance of Equation (7) is the same for small and large systems, we see by using Equations (6) and (10) that the Euler equation for a small system takes a different form

(11) U=TS+γΩ+μN+E

which shows the central role of the subdivision potential. Equation (7) together with Equation (8) gives

(12) dE=−SdT−Ωdγ−Ndμ

Using the Gibbs-Duhem equation in the large surface area (thermodynamic) limit it follows that E=0 in this limit. This implies that $\hat{\gamma }=\gamma$ in the thermodynamic limit.

Equation (12) shows furthermore that

(13) $${\left(\frac{\partial E}{\partial T}\right)}_{\gamma ,\mu }=-S,\;\;\;{\left(\frac{\partial E}{\partial \gamma }\right)}_{T,\mu }=\Omega ,\;\;\;{\left(\frac{\partial E}{\partial \mu }\right)}_{T,\gamma }=-N$$

The intensive variables *T*, γ, μ determine all the extensive variables *S*, Ω, *N*. This is possible for a small sphere, and is a feature specific to small systems. In the large sphere limit *S*, Ω, *N* all become infinitely large. The change in the subdivision potential may be written in a form more appropriate for the environment the small system is in

(14) $$dE=\Omega^{2}{\left(\frac{\partial s}{\partial \Omega }\right)}_{T,\mu }\;dT-\Omega {\left(\frac{\partial \gamma }{\partial \Omega }\right)}_{T,\mu }\;d\Omega +\Omega^{2}{\left(\frac{\partial n}{\partial \Omega }\right)}_{T,\mu }\;d\mu$$

The effects of size on intensive variables, a characteristic feature of small systems, are now directly available as the differential coefficients of Equation (14). This relation is especially useful because the independent variables are the environment variables.

## 3. The Model

The one-particle canonical partition function for a small sphere with surface adsorption follows from statistical mechanics [7]:

(15) $$Q_{1}\left(T,\Omega \right)=\frac{\Omega }{\Lambda^{2}}\exp\left(-\beta U^{s}\right)$$

where $U^{s}$ is the potential energy of interaction between the adsorbent and an adsorbed molecule, $\Lambda \equiv \sqrt{h^{2}/\left(2\pi mk_{B}T\right)}$ is the mean thermal de Broglie wave length. Here *m* is the particle mass. The *N*-particle canonical partition function becomes:

(16) $$Q\left(T,\Omega ,N\right)=\frac{1}{N!}Q_{1}^{N}\left(T,\Omega \right)$$

The grand canonical partition function equals

(17) $$\begin{array}{cc} \Xi \left(T,\Omega ,\mu \right) & =\sum_{N=0}^{\infty }\exp\left(\beta \mu N\right)Q\left(T,\Omega ,N\right)\\ & =\exp\left(\exp\left(\beta \mu \right)Q_{1}\left(T,\Omega \right)\right)\\ & =\exp\left\{\frac{\Omega }{\Lambda^{2}}\exp\left[\beta \left(\mu -U^{s}\right)\right]\right\} \end{array}$$

where we used Equation (16). By introducing the expressions above, thermodynamic properties can be derived in terms of *T*, Ω, μ. From Equation (17) we find for the integral surface tension

(18) $$\hat{\gamma }=-\frac{k_{B}T}{\Omega }\ln\Xi \left(T,\Omega ,\mu \right)=-\frac{k_{B}T}{\Lambda^{2}}\exp\left[\beta \left(\mu -U^{s}\right)\right]$$

This is the equation of state for the adsorbed phase controlled by the grand canonical ensemble. The differential surface tension is given by Equation (9)

(19) $$\gamma =-\frac{k_{B}T}{\Lambda^{2}}\exp\left[\beta \left(\mu -U^{s}\right)\right]\left(1-\beta \Omega {\left(\frac{\partial U^{s}}{\partial \Omega }\right)}_{T,\mu }\right)$$

By using Equation (10), we can now determine the subdivision potential:

(20) $$E=-\left(\frac{\Omega }{\Lambda^{2}}\right)\exp\left[\beta \left(\mu -U^{s}\right)\right]\Omega {\left(\frac{\partial U^{s}}{\partial \Omega }\right)}_{T,\mu }$$

In the thermodynamic limit $U^{s}$ becomes independent of Ω so that E  approaches zero. The entropy density s=S/Ω becomes using Equation (9)

(21) $$s=\left(k_{B}/\Lambda^{2}\right)\exp\left[\beta \left(\mu -U^{s}\right)\right]\left[2-\beta \left(\mu -U^{s}\right)\right]$$

The particle density n=N/Ω becomes using Equation (9)

(22) $$n=\left(1/\Lambda^{2}\right)\exp\left[\beta \left(\mu -U^{s}\right)\right]$$

Thermodynamic quantities of the adsorbed phase may be expressed per molecule. The quantities are then given by particularly simple expressions. It follows from Equations (18)–(22) that

(23) $$\frac{\hat{\gamma }\Omega }{N}=-k_{B}T$$

(24) $$\frac{\gamma \Omega }{N}=-k_{B}T+\Omega {\left(\frac{\partial U^{s}}{\partial \Omega }\right)}_{T,\mu }$$

(25) $$\frac{E}{N}=-\Omega {\left(\frac{\partial U^{s}}{\partial \Omega }\right)}_{T,\mu }$$

(26) $$\frac{S}{N}=k_{B}\left[2-\beta \left(\mu -U^{s}\right)\right]$$

(27) $$\frac{U}{N}=\frac{X}{N}+T\frac{S}{N}+\mu =-k_{B}T+k_{B}T\left[2-\beta \left(\mu -U^{s}\right)\right]+\mu =k_{B}T+U^{s}$$

For the differential entropy and internal energy we have the model expressions

(28) $$\begin{array}{cc} {\left(\frac{\partial S}{\partial N}\right)}_{T,\Omega } & ={\left(\frac{\partial s}{\partial \mu }\right)}_{T,\Omega }/{\left(\frac{\partial n}{\partial \mu }\right)}_{T,\Omega }\\ & =k_{B}\left[1-\beta \left(\mu -U^{s}\right)\right] \end{array}$$

(29) $${\left(\frac{\partial U}{\partial N}\right)}_{T,\Omega }=k_{B}T+U^{s}$$

## 4. Correspondence with Experiment

Although comparisons with experimental results are not part of this work, the reader may be interested in the relevant relations. Furthermore, the connection to experiment may help make the description less abstract, so we allow ourselves this small detour here. We are mainly interested in how the thermodynamic properties of the adsorbed phase are affected when we vary Ω. The experimental system is typically a large collection of spherical adsorbents, such as a powder, in equilibrium with the adsorbate gas. From an experimental perspective, by using the environment variable Ω we imply that the small system is rigid. This is because we do not have any direct means of controlling the adsorbent size. We control the system experimentally through the surrounding gas. Thus, if the adsorbent is not rigid, we cannot prevent the adsorbent size from fluctuating. Instead we treat Ω as a variable parameter, and we control Ω by performing experimental measurements on (monodisperse) samples prepared with different values of Ω.

In order to assess the statistical model we may derive relations connecting thermodynamic properties of the adsorbed phase to experimentally convenient variables, see [8,9], and Appendix A. We may asses the adsorbed phase entropy and energy per molecule, and the differential entropy and energy (all relative to the gas) by

(30) $$\frac{S}{N}-s^{G}=-kT{\left(\frac{\partial \ln p}{\partial T}\right)}_{\hat{\gamma },\Omega }$$

(31) $$\frac{U}{N}-u^{G}=kT\left[1-T{\left(\frac{\partial \log p}{\partial T}\right)}_{\frac{\hat{\gamma }}{T},\Omega }\right]$$

(32) $${\left(\frac{\partial S}{\partial N}\right)}_{T,\Omega }-s^{G}=-kT{\left(\frac{\partial \ln p}{\partial T}\right)}_{\Omega ,N}$$

(33) $${\left(\frac{\partial U}{\partial N}\right)}_{T,\Omega }-u^{G}=kT\left[1-T{\left(\frac{\partial \log p}{\partial T}\right)}_{\Omega ,N}\right]$$

where $s^{G}\equiv S^{G}/N^{G}$ is the gas entropy $S^{G}$ per gas molecule $N^{G}$, and $u^{G}\equiv U^{G}/N^{G}$ is the gas internal energy $U^{G}$ per gas molecule. These are only a selection of relations that may be useful.

## 5. The Potential $U^{s}$

**Figure 1:**
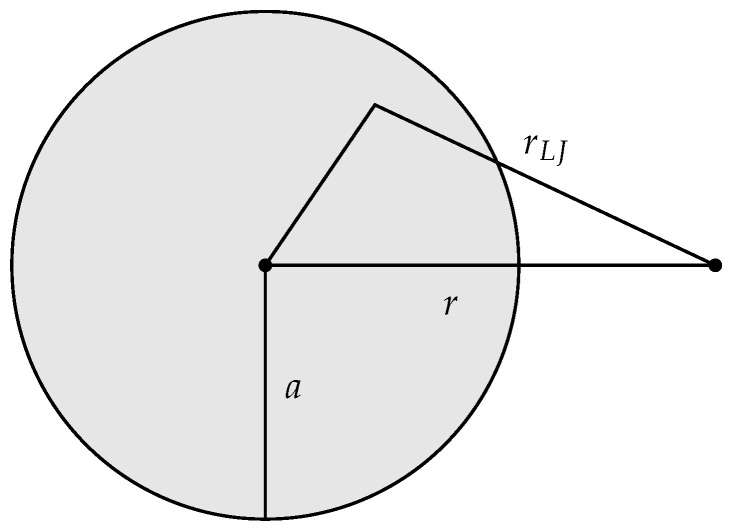
Illustration of the adsorbent with radius *a*. The distance between a volume element of the adsorbent and a gas molecule is given by $r_{LJ}$, and the distance between the adsorbent center and the same gas molecule is given by *r*.

The adsorbent functioning as an external field was represented by a sphere of uniform density ρ and radius *a*, see Figure 1. The total interaction energy U was determined by integrating the interaction energy $4\pi \rho u\left(r_{LJ}\right)a^{\prime 2}\;da^{\prime }$ between a volume element of the adsorbent, and a gas molecule separated by the distance $r_{LJ}$. The interaction potential $u(r_{LJ})$ was given by the standard Lennard-Jones 12-6 potential:

(34) $$u\left(r_{LJ}\right)=4\epsilon \left[{\left(\frac{\sigma }{r_{LJ}}\right)}^{12}-{\left(\frac{\sigma }{r_{LJ}}\right)}^{6}\right]$$

where ϵ is the energy parameter of the interaction, and σ is the length parameter of the interaction. We used reduced units; ϵ as the unit of energy, σ as the unit of length, the gas molecular mass as the unit of mass, and $k_{B}=1$.

Integrating Equation (34) over the spherical adsorbent we have

(35) $$U\left(a,r\right)=\frac{16\pi \epsilon \rho \sigma^{3}}{3}\left[\frac{\left(15a^{3}r^{6}+63a^{5}r^{4}+45a^{7}r^{2}+5a^{9}\right)\sigma^{9}}{15{\left(r^{2}-a^{2}\right)}^{9}}-\frac{a^{3}\sigma^{3}}{{\left(r^{2}-a^{2}\right)}^{3}}\right],\;r>a$$

where *r* is the center to center distance between the adsorbent and a gas molecule. The location of an adsorbed molecule relative to the center of the adsorbent is *R*, which is determined by the control variable Ω by the equation $\Omega =4\pi R^{2}$. Operationally it is more practical to control *a* and determine Ω by a dividing surface condition involving *a*, than it is the other way around. The correspondence between *R* and *a* is established by the condition U(a,r=R)=min[U(a,r)], i.e., for a given adsorbent size *a*, the location *R* of the dividing surface is the location of the minimum of the potential U. Thus, the adsorption energy $U^{s}$ of an adsorbed molecule is determined by *a*, Equation (35), the fixed chosen condition, and a fixed value of ρ. We choose $\rho =1/\left(\left[4\pi {\left(\sigma /2\right)}^{3}\right]/3\right)$.

## 6. Results

In this section we present calculations for the ideal adsorbed phase to show the size dependence of some important intensive properties, and give some substance to the thermodynamic framework.

**Figure 2:**
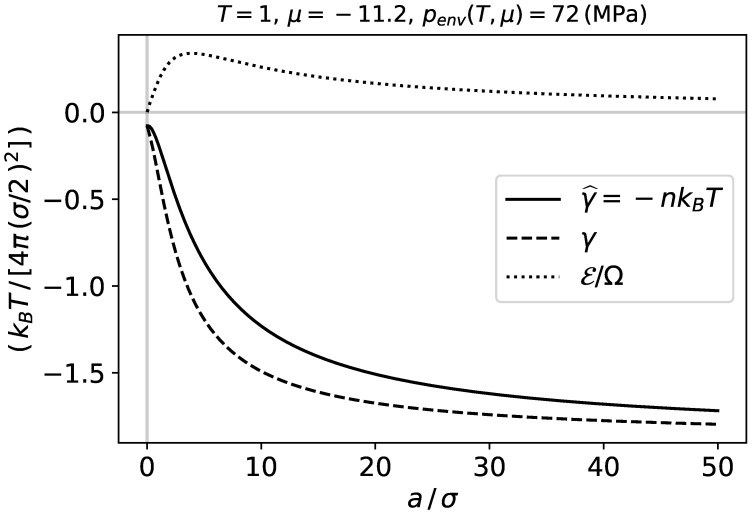
Adsorbed phase thermodynamic quantities per unit area at constant temperature and chemical potential. The figure shows that when the system is small the characteristic energy of the adsorbed phase per unit area depends on the size of the adsorbent. The transition from small to macroscopic is continuous and may reasonably be considered to be beyond 50 σ for this system.

Figure 2 shows the integral surface tension $\hat{\gamma }$ which is the characteristic energy per unit area, the differential surface tension γ, and the subdivision potential per unit area E/Ω, as functions of the adsorbent radius *a* at constant temperature and chemical potential. The names integral and differential are here used to refer to the relation between $\hat{\gamma }$ and γ in Equation (9). The quantities $\hat{\gamma }$, γ and E are calculated by Equations (18)–(20). We observe that when the system becomes larger, E approaches zero, and $\hat{\gamma }$ and γ both approach a plateau value.

**Figure 3:**
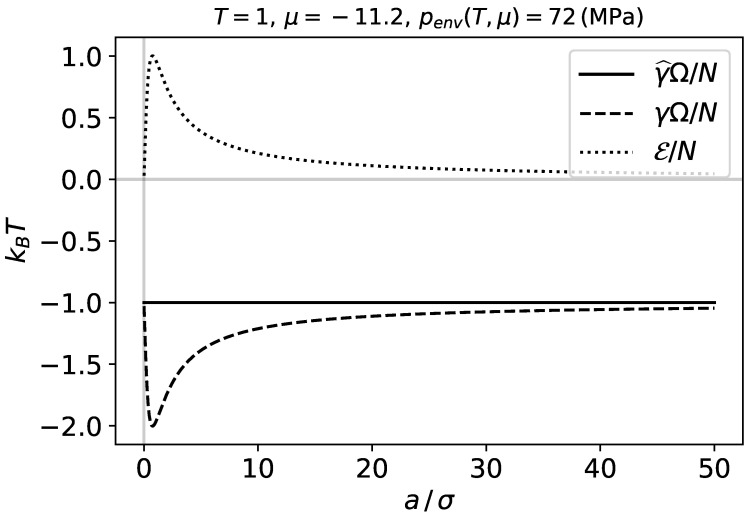
Adsorbed phase thermodynamic quantities per molecule at constant temperature and chemical potential.

Figure 3 shows the characteristic energy per molecule, the energy γΩ per molecule, and the subdivision potential per molecule E/N, as functions of the adsorbent radius *a* at constant temperature and chemical potential. We observe that when the system becomes larger, E approaches zero, and γ approaches the limit value $-k_{B}T$. Here, the characteristic energy per molecule follows the 2-dimensional analogue of the ideal gas law $\hat{\gamma }\Omega =-nk_{B}T$.

## 7. Discussion

As we are considering a two-dimensional ideal gas, or a dilute adsorbed phase with free mobility, we expect the phase to follow the two-dimensional analogue of the ideal gas law $\hat{\gamma }=-nk_{B}T$. This is consistent with Equation (23) and Figure 3. When the adsorbent size *a* approaches zero, the dividing surface radius *R* should approach the potential minimum distance of the interaction between a single adsorbent atom and an adsorbed molecule. This distance is $2^{1/6}\sigma$ for the Lennard-Jones potential. This is because of the way we have defined *R*.

In the macroscopic limit the energy γΩ is a linear function of Ω. The tension γ then becomes equal to the characteristic energy per unit area of the adsorbed phase, i.e., γ=(U−TS−μN)/Ω, for the environment *T*, Ω, μ. When the system is small γΩ deviates from the characteristic energy by E, as shown by Equation (12). The integral surface tension was therefore defined to represent this important quantity, i.e., $\hat{\gamma }=\left(U-TS-\mu N\right)/\Omega =X/\Omega$. The tension γ is now given by the relation γ=∂X/∂Ω. This relation is always valid whether the system is small or large. It is only in the special case of the macroscopic limit that the relation ∂X/∂Ω=X/Ω is true. The integral and differential surface tensions are then equal. This way of stating the smallness is expressed by Equations (9) and (10), which may be rewritten as

(36) $$\frac{\hat{\gamma }\Omega }{\Omega }-{\left(\frac{\partial \hat{\gamma }\Omega }{\partial \Omega }\right)}_{T,\mu }=\frac{X}{\Omega }-{\left(\frac{\partial X}{\partial \Omega }\right)}_{T,\mu }=\frac{E}{\Omega }$$

where E goes to zero in the macroscopic limit. Both $\hat{\gamma }$ and γ are functions of the system size, thus E measures the difference expressed by Equation (36), and not simply the difference between the characteristic energy and the limit of γΩ.

The effects of size on intensive variables, characteristic of small systems, may be expressed by the subdivision potential E and its derivatives, according to Equation (14). The physical significance of E is more clear if we use the definitions $E\equiv (\hat{\gamma }-\gamma )\Omega =X-\gamma \Omega$ and $\Omega_{t}\equiv N\Omega$ to rewrite Equation (1) as

(37) $$dU_{t}=T\;dS_{t}+\gamma \;d\Omega_{t}+\mu \;dN_{t}+E\;dN$$

An alternative definition of E is then

(38) $$E\equiv {\left(\frac{\partial U_{t}}{\partial N}\right)}_{S_{t},\Omega_{t},N_{t}}=-\frac{\Omega }{N}{\left(\frac{\partial U_{t}}{\partial \Omega }\right)}_{S_{t},\Omega_{t},N_{t}}$$

By this definition, we see that E is the work required to increase the number of replicas while keeping $S_{t}$, $\Omega_{t}$, and $N_{t}$ constant. Since the total surface area is constant, it must be redistributed across the new number of replicas. The area of each replica therefore becomes smaller, which for a fixed shape means larger curvature. Thus, the subdivision potential for the given system, with fixed shape, is also the work required to change the adsorbed phase curvature while keeping $S_{t}$, $\Omega_{t}$, and $N_{t}$ constant. If the process of adding a system to the ensemble is at constant Ω instead of $\Omega_{t}$ the work is given by $X=\hat{\gamma }\Omega =\gamma \Omega +E$.

When the intensive properties become independent of the curvature E=0, which is consistent with Equations (9) and (10). This occurs in the macroscopic limit, when the adsorbent becomes large, which is consistent with Figure 2 and Figure 3. All the differential coefficients, expressing dependence of intensive properties on curvature, are then zero, and dE=0 by Equation (14). It also follows from these figures that *a* must be larger than 50σ for E to become small.

## 8. Concluding Remarks

The above analysis shows that we can use the adsorbed phase as a small thermodynamic system in the sense of Hill. The analysis for our ideal adsorbed gas model becomes very simple. This allows the close relationship between the subdivision potential and the dependence of intensive properties on size, and the internal structure of nanothermodynamics to be seen more clearly.

## Appendix A

The Gibbs-Duhem relation for a single component homogeneous phase α is given by

(A1) $$d\mu^{\alpha }=-s^{\alpha }\;dT^{\alpha }+v^{\alpha }\;dp^{\alpha }$$

where $s^{\alpha }\equiv S^{\alpha }/N^{\alpha }$ is the entropy per molecule, and $v^{\alpha }\equiv V^{\alpha }/N^{\alpha }$ is the volume per molecule. At chemical equilibrium the chemical potential μ of the adsorbed phase, and the chemical potential $\mu^{G}$ of the gas phase are subject to the relations $\mu =\mu^{G}$ and $d\mu =d\mu^{G}$. The Gibbs-Duhem relation for the ideal gas may then be written as

(A2) $$d\mu =-s^{G}\;dT+kT\;d\log p$$

where *p* is the equilibrium gas pressure, *T* is the equilibrium temperature, and superscript *G* denotes the gas phase.

Using Equations (6), (8) and (A2) we have

(A3) $$\begin{array}{cc} -(dX)/N-(S/N)\;dT+(\gamma /N)\;d\Omega & =-s^{G}\;dT+kT\;d\log p\\ \frac{S}{N}-s^{G} & =-kT{\left(\frac{\partial \log p}{\partial T}\right)}_{X,\Omega }\\ & =-kT{\left(\frac{\partial \log p}{\partial T}\right)}_{\hat{\gamma },\Omega } \end{array}$$

From Equation (8) and the definition $F\equiv \hat{\gamma }\Omega +\mu N$ we have

(A4) dF=−S dT+γ dΩ+μ dN

From this equation we obtain the Maxwell relation

(A5) $${\left(\frac{\partial \mu }{\partial T}\right)}_{\Omega ,N}=-{\left(\frac{\partial S}{\partial N}\right)}_{T,\Omega }$$

For variations in the adsorbed phase chemical potential at constant Ω and *N*, at equilibrium with the gas, we then have

(A6) $$\begin{array}{cc} d\mu ={\left(\frac{\partial \mu }{\partial T}\right)}_{\Omega ,N}\;dT & =-{\left(\frac{\partial S}{\partial N}\right)}_{T,\Omega }\;dT=-s^{G}\;dT+kT\;d\log p\\ {\left(\frac{\partial S}{\partial N}\right)}_{T,\Omega }-s^{G} & =-kT{\left(\frac{\partial \log p}{\partial T}\right)}_{\Omega ,N} \end{array}$$

From Equation (6) and the equilibrium condition $\mu =\mu^{G}$ we have

(A7) $$\begin{array}{cc} \frac{U-TS-X}{N} & =\frac{U^{G}-TS^{G}+pV^{G}}{N^{G}}\\ & =u^{G}-Ts^{G}+kT \end{array}$$

where $u^{G}\equiv U^{G}/N^{G}$ is the gas internal energy per gas molecule. We also have from Equation (8), using Equation (A7) to eliminate X/(TN):

(A8) $$\begin{array}{cc} d\mu & =-\frac{1}{N}\;dX-\frac{S}{N}\;dT+\frac{\gamma }{N}\;d\Omega \\ & =-\frac{1}{N}\;d\left(TX/T\right)-\frac{S}{N}\;dT+\frac{\gamma }{N}\;d\Omega \\ & =-\frac{T}{N}\;d\left(X/T\right)-\left(\frac{X}{TN}+\frac{S}{N}\right)\;dT+\frac{\gamma }{N}\;d\Omega \\ & =-\left(\frac{X}{TN}+\frac{S}{N}\right)\;dT,\;\left(\frac{X}{T},\Omega \;\mathrm{const}.\right)\\ & =\frac{u^{G}-Ts^{G}+kT-U/N}{T}\;dT,\;\left(\frac{X}{T},\Omega \;\mathrm{const}.\right) \end{array}$$

Using $d\mu =d\mu^{G}$ and Equation (A8) we obtain

(A9) $$\begin{array}{cc} \frac{u^{G}-Ts^{G}+kT-U/N}{T}\;dT & =-s^{G}\;dT+kT\;d\log p\\ \frac{U}{N}-u^{G} & =kT\left[1-T{\left(\frac{\partial \log p}{\partial T}\right)}_{\frac{X}{T},\Omega }\right] \end{array}$$

Using Equations (7), (A2), (A4) and (A7) we have

(A10) $$\begin{array}{cc} -{\left(\frac{\partial S}{\partial N}\right)}_{T,\Omega } & =-\frac{1}{T}{\left(\frac{\partial U}{\partial N}\right)}_{T,\Omega }+\frac{\mu }{T}\\ & =-\frac{1}{T}{\left(\frac{\partial U}{\partial N}\right)}_{T,\Omega }+\frac{u^{G}-Ts^{G}+kT}{T}\\ -s^{G}\;dT+kT\;d\log p & =\left[-\frac{1}{T}{\left(\frac{\partial U}{\partial N}\right)}_{T,\Omega }+\frac{u^{G}-Ts^{G}+kT}{T}\right]\;dT\\ kT{\left(\frac{\partial \log p}{\partial T}\right)}_{\Omega ,N} & =-\frac{1}{T}\left[{\left(\frac{\partial U}{\partial N}\right)}_{T,\Omega }-u^{G}-kT\right]\\ {\left(\frac{\partial U}{\partial N}\right)}_{T,\Omega }-u^{G} & =kT\left[1-T{\left(\frac{\partial \log p}{\partial T}\right)}_{\Omega ,N}\right] \end{array}$$
